# Supplementary material for: Subnational tuberculosis burden estimation for Pakistan
Source: PLOS Glob Public Health. 2024 Sep 23;4(9):e0003653. doi: 10.1371/journal.pgph.0003653 (PMC11419375; doi:10.1371/journal.pgph.0003653)
Supplement: S1 Text — (DOCX) [file pgph.0003653.s001.docx]

**Subnational tuberculosis burden estimation for Pakistan**

Alvaro Schwalb^1,2,3^, Zia Samad^4^, Aashifa Yaqoob^4^, Razia Fatima^4^, Rein M.G.J. Houben^1,2^

*^1^TB Modelling Group, TB Centre, London School of Hygiene and Tropical Medicine, London, United Kingdom; ^2^Department of Infectious Disease Epidemiology, London School of Hygiene and Tropical Medicine, London, United Kingdom; ^3^Instituto de Medicina Tropical Alexander von Humboldt, Universidad Peruana Cayetano Heredia, Lima, Peru; ^4^Common Management Unit for AIDS, TB & Malaria, Ministry of National Health Services, Regulations & Coordination, Islamabad, Pakistan.*

**Correspondence:** Alvaro Schwalb, Department of Infectious Disease Epidemiology, London School of Hygiene and Tropical Medicine, Keppel St, London, WC1E 7HT United Kingdom ([alvaro.schwalb@lshtm.ac.uk](mailto:alvaro.schwalb@lshtm.ac.uk))

**GitHub:** https://github.com/aschwalbc/SUBsET-PAK

# S1 Text

## District divisions of Pakistan

Annually, new districts are established in Pakistan; however, TB notification updates from the NTP take time to reflect these changes, resulting in TB notifications being reported as in previous years. To ensure consistency, the map of districts was adjusted to align with the NTP's TB notification reporting, necessitating some district merges:

- *Sibi [BA]:* Lehri was merged into Sibi
- *Killa Abdulah [BA]:* Chaman was merged into Killa Abdulah
- *Khizer [GB]:* Gupis-Yasin was merged into Khizer
- *Diamer [GB]:* Darel and Tangir were merged into Diamer
- *Skardu [GB]:* Rondu was merged into Skardu
- *Mansehra [KP]:* Torghar was merged into Mansehra
- *Chitral [KP]:* Chital Upper and Chitral Lower were merged into Chitral
- *Kohistan [KP]:* Kolai Palas, Upper and Lower Kohistan were merged into Kohistan
