# Supplementary material for: Subnational tuberculosis burden estimation for Pakistan
Source: PLOS Glob Public Health. 2024 Sep 23;4(9):e0003653. doi: 10.1371/journal.pgph.0003653 (PMC11419375; doi:10.1371/journal.pgph.0003653)
Supplement: S2 Text — (DOCX) [file pgph.0003653.s002.docx]

**Subnational tuberculosis burden estimation for Pakistan**

Alvaro Schwalb^1,2,3^, Zia Samad^4^, Aashifa Yaqoob^4^, Razia Fatima^4^, Rein M.G.J. Houben^1,2^

*^1^TB Modelling Group, TB Centre, London School of Hygiene and Tropical Medicine, London, United Kingdom; ^2^Department of Infectious Disease Epidemiology, London School of Hygiene and Tropical Medicine, London, United Kingdom; ^3^Instituto de Medicina Tropical Alexander von Humboldt, Universidad Peruana Cayetano Heredia, Lima, Peru; ^4^Common Management Unit for AIDS, TB & Malaria, Ministry of National Health Services, Regulations & Coordination, Islamabad, Pakistan.*

**Correspondence:** Alvaro Schwalb, Department of Infectious Disease Epidemiology, London School of Hygiene and Tropical Medicine, Keppel St, London, WC1E 7HT United Kingdom ([alvaro.schwalb@lshtm.ac.uk](mailto:alvaro.schwalb@lshtm.ac.uk))

**GitHub:** https://github.com/aschwalbc/SUBsET-PAK

# S2 Text

## Data completeness

Data for TB notification, population, and healthcare facilities were available for all districts. For selected variables, if district values were missing (BA: Chagai, Jhal Magsi, Musakhel, Panjgur, and Zhob), regional averages were used. Information on the level of urbanisation, households with one room, and food insecurity was not available for AJK and GB at the district or regional level. Therefore, for AJK and GB, the same incidence is estimated in each district as no district-specific weight scores could be assigned. Furthermore, there was no information on households with one room and food insecurity for ICT; however, since ICT consists of a single administrative unit the weight score is unaffected.
