# Supplementary material for: Subnational tuberculosis burden estimation for Pakistan
Source: PLOS Glob Public Health. 2024 Sep 23;4(9):e0003653. doi: 10.1371/journal.pgph.0003653 (PMC11419375; doi:10.1371/journal.pgph.0003653)
Supplement: S1 Table — (DOCX) [file pgph.0003653.s003.docx]

**Subnational tuberculosis burden estimation for Pakistan**

Alvaro Schwalb^1,2,3^, Zia Samad^4^, Aashifa Yaqoob^4^, Razia Fatima^4^, Rein M.G.J. Houben^1,2^

*^1^TB Modelling Group, TB Centre, London School of Hygiene and Tropical Medicine, London, United Kingdom; ^2^Department of Infectious Disease Epidemiology, London School of Hygiene and Tropical Medicine, London, United Kingdom; ^3^Instituto de Medicina Tropical Alexander von Humboldt, Universidad Peruana Cayetano Heredia, Lima, Peru; ^4^Common Management Unit for AIDS, TB & Malaria, Ministry of National Health Services, Regulations & Coordination, Islamabad, Pakistan.*

**Correspondence:** Alvaro Schwalb, Department of Infectious Disease Epidemiology, London School of Hygiene and Tropical Medicine, Keppel St, London, WC1E 7HT United Kingdom ([alvaro.schwalb@lshtm.ac.uk](mailto:alvaro.schwalb@lshtm.ac.uk))

**GitHub:** https://github.com/aschwalbc/SUBsET-PAK

# S1 Table

## District estimates of TB burden and case detection rate in Pakistan.

| **ID** | **Region** | **District** | **TB incidence per 100k**  **(95%CI)** | **Case detection rate**  **(95%CI)** |
| --- | --- | --- | --- | --- |
| PK101 | AJK | Bagh | 243 (177 – 320) | 61% (46 – 83) |
| PK102 | AJK | Bhimber | 243 (177 – 320) | 66% (50 – 91) |
| PK103 | AJK | Jhelum Vellay | 243 (177 – 320) | 45% (34 – 61) |
| PK104 | AJK | Haveli | 243 (177 – 320) | 41% (31 – 57) |
| PK105 | AJK | Kotli | 243 (177 – 320) | 32% (24 – 44) |
| PK106 | AJK | Mirpur | 243 (177 – 320) | 42% (32 – 57) |
| PK107 | AJK | Muzaffarabad | 243 (177 – 320) | 49% (37 – 67) |
| PK108 | AJK | Neelum | 243 (177 – 320) | 32% (25 – 44) |
| PK109 | AJK | Poonch | 243 (177 – 320) | 29% (22 – 39) |
| PK110 | AJK | Sudhnoti | 243 (177 – 320) | 36% (28 – 50) |
| PK201 | BA | Awaran | 275 (200 – 361) | 15% (12 – 21) |
| PK202 | BA | Barkhan | 219 (160 – 288) | 15% (11 – 20) |
| PK203 | BA | Chagai | 190 (138 – 249) | 26% (20 – 35) |
| PK204 | BA | Dera Bugti | 187 (136 – 246) | 10% (8 – 14) |
| PK205 | BA | Gwadar | 111 (81 – 146) | 17% (13 – 23) |
| PK206 | BA | Harnai | 110 (80 – 145) | 15% (11 – 20) |
| PK207 | BA | Jaffarabad | 287 (209 – 378) | 47% (36 – 64) |
| PK208 | BA | Jhal Magsi | 191 (139 – 251) | 187% (142 – 257) |
| PK209 | BA | Kachhi | 210 (153 – 277) | 13% (10 – 18) |
| PK210 | BA | Kalat | 197 (144 – 259) | 6% (5 – 9) |
| PK211 | BA | Kech/Turbat | 159 (116 – 209) | 13% (10 – 18) |
| PK212 | BA | Kharan | 145 (106 – 191) | 46% (35 – 63) |
| PK213 | BA | Khuzdar | 243 (177 – 320) | 21% (16 – 29) |
| PK214 | BA | Killa Abdullah | 180 (131 – 237) | 26% (20 – 36) |
| PK215 | BA | Killa Saifullah | 160 (116 – 210) | 84% (64 – 116) |
| PK216 | BA | Kohlu | 132 (96 – 173) | 20% (15 – 27) |
| PK217 | BA | Lasbela | 138 (100 – 181) | 63% (48 – 87) |
| PK219 | BA | Loralai | 157 (115 – 207) | 36% (28 – 50) |
| PK220 | BA | Mastung | 169 (123 – 222) | 25% (19 – 34) |
| PK221 | BA | Musakhel | 189 (138 – 249) | 9% (7 – 12) |
| PK222 | BA | Nasirabad | 277 (201 – 363) | 33% (25 – 45) |
| PK223 | BA | Nushki | 138 (101 – 182) | 72% (55 – 99) |
| PK224 | BA | Panjgur | 178 (129 – 233) | 13% (10 – 18) |
| PK225 | BA | Pishin | 144 (105 – 190) | 47% (36 – 65) |
| PK226 | BA | Quetta | 145 (106 – 191) | 68% (51 – 93) |
| PK227 | BA | Sherani | 120 (88 – 158) | 52% (39 – 71) |
| PK228 | BA | Sibi | 176 (128 – 232) | 91% (69 – 125) |
| PK229 | BA | Sohbatpur | 324 (236 – 426) | 14% (11 – 19) |
| PK230 | BA | Washuk | 158 (115 – 208) | 13% (10 – 18) |
| PK231 | BA | Zhob | 184 (134 – 242) | 63% (48 – 87) |
| PK232 | BA | Ziarat | 142 (104 – 187) | 8% (6 – 11) |
| PK233 | BA | Shaheed Sikandar Abad | 240 (175 – 315) | 9% (7 – 12) |
| PK234 | BA | Duki | 148 (108 – 195) | 16% (12 – 23) |
| PK301 | GB | Astore | 162 (118 – 213) | 50% (38 – 69) |
| PK302 | GB | Diamir | 162 (118 – 213) | 288% (219 – 395) |
| PK303 | GB | Ghanche | 162 (118 – 213) | 9% (7 – 12) |
| PK304 | GB | Khizer | 162 (118 – 213) | 23% (17 – 31) |
| PK305 | GB | Gilgit | 162 (118 – 213) | 118% (90 – 162) |
| PK306 | GB | Hunza | 162 (118 – 213) | 12% (9 – 16) |
| PK307 | GB | Skardu | 162 (118 – 213) | 71% (54 – 98) |
| PK308 | GB | Nagar | 162 (118 – 213) | 11% (8 – 15) |
| PK309 | GB | Kharmang | 162 (118 – 213) | 6% (5 – 8) |
| PK310 | GB | Shigar | 162 (118 – 213) | 16% (12 – 22) |
| PK401 | ICT | Islamabad | 230 (168 – 303) | 26% (20 – 36) |
| PK501 | KP | Abbottabad | 256 (186 – 336) | 40% (31 – 55) |
| PK502 | KP | Bajaur | 322 (235 – 424) | 8% (6 – 11) |
| PK503 | KP | Bannu | 303 (221 – 398) | 46% (35 – 63) |
| PK504 | KP | Batagram | 367 (267 – 482) | 23% (18 – 32) |
| PK505 | KP | Buner | 307 (224 – 404) | 15% (12 – 21) |
| PK506 | KP | Charsadda | 323 (236 – 425) | 26% (20 – 35) |
| PK507 | KP | Chitral | 269 (196 – 353) | 18% (14 – 25) |
| PK509 | KP | Dera Ismail Khan | 216 (157 – 283) | 46% (35 – 64) |
| PK510 | KP | Hangu | 237 (173 – 312) | 74% (56 – 101) |
| PK511 | KP | Haripur | 281 (205 – 369) | 30% (22 – 41) |
| PK512 | KP | Karak | 245 (178 – 322) | 35% (27 – 48) |
| PK513 | KP | Khyber | 229 (167 – 301) | 23% (18 – 32) |
| PK514 | KP | Kohat | 264 (193 – 348) | 47% (36 – 65) |
| PK517 | KP | Kohistan | 332 (241 – 436) | 10% (8 – 14) |
| PK518 | KP | Kurram | 264 (192 – 348) | 23% (17 – 31) |
| PK519 | KP | Lakki Marwat | 267 (194 – 351) | 56% (43 – 77) |
| PK520 | KP | Lower Dir | 318 (232 – 418) | 14% (10 – 19) |
| PK521 | KP | Malakand | 268 (195 – 353) | 32% (24 – 43) |
| PK522 | KP | Mansehra | 268 (195 – 352) | 28% (22 – 39) |
| PK523 | KP | Mardan | 296 (215 – 389) | 27% (20 – 37) |
| PK524 | KP | Mohmand | 340 (248 – 447) | 12% (9 – 16) |
| PK525 | KP | North Waziristan | 273 (199 – 359) | 22% (16 – 30) |
| PK526 | KP | Nowshera | 263 (191 – 345) | 26% (20 – 35) |
| PK527 | KP | Orakzai | 258 (188 – 339) | 42% (32 – 58) |
| PK528 | KP | Peshawar | 202 (147 – 265) | 74% (56 – 101) |
| PK529 | KP | Shangla | 249 (181 – 327) | 43% (33 – 59) |
| PK530 | KP | South Waziristan | 332 (242 – 437) | 12% (9 – 17) |
| PK531 | KP | Swabi | 257 (187 – 338) | 24% (18 – 33) |
| PK532 | KP | Swat | 249 (181 – 327) | 24% (18 – 33) |
| PK533 | KP | Tank | 321 (234 – 421) | 41% (31 – 56) |
| PK535 | KP | Upper Dir | 315 (229 – 414) | 8% (6 – 11) |
| PK601 | PB | Attock | 187 (136 – 246) | 71% (54 – 97) |
| PK602 | PB | Bahawalnagar | 295 (215 – 388) | 73% (56 – 101) |
| PK603 | PB | Bahawalpur | 287 (209 – 377) | 69% (53 – 95) |
| PK604 | PB | Bhakkar | 277 (202 – 365) | 76% (58 – 105) |
| PK605 | PB | Chakwal | 212 (154 – 279) | 75% (57 –103) |
| PK606 | PB | Chiniot | 256 (187 – 337) | 80% (60 –109) |
| PK607 | PB | Dera Ghazi Khan | 297 (216 – 390) | 81% (62 – 111) |
| PK608 | PB | Faisalabad | 208 (151 – 273) | 75% (57 – 103) |
| PK609 | PB | Gujranwala | 194 (141 – 255) | 97% (74 – 133) |
| PK610 | PB | Gujrat | 180 (131 – 237) | 54% (41 – 75) |
| PK611 | PB | Hafizabad | 214 (156 – 281) | 82% (62 – 113) |
| PK612 | PB | Jhang | 289 (211 – 380) | 76% (58 – 104) |
| PK613 | PB | Jhelum | 227 (166 – 299) | 105% (80 – 144) |
| PK614 | PB | Kasur | 326 (237 – 428) | 67% (51 – 91) |
| PK615 | PB | Khanewal | 305 (222 – 401) | 61% (46 – 83) |
| PK616 | PB | Khushab | 223 (162 – 293) | 106% (80 – 145) |
| PK617 | PB | Lahore | 200 (146 – 263) | 72% (54 – 98) |
| PK618 | PB | Leiah | 317 (231 – 416) | 45% (34 – 62) |
| PK619 | PB | Lodhran | 287 (209 – 377) | 58% (44 – 80) |
| PK620 | PB | Mandi Bahauddin | 214 (156 – 282) | 114% (87 – 156) |
| PK621 | PB | Mianwali | 244 (178 – 321) | 96% (73 – 132) |
| PK622 | PB | Multan | 244 (178 – 321) | 80% (61 –110) |
| PK623 | PB | Muzaffargarh | 337 (245 – 443) | 62% (47 – 85) |
| PK624 | PB | Nankana Sahib | 235 (171 – 309) | 104% (79 – 143) |
| PK625 | PB | Narowal | 234 (170 – 307) | 50% (38 – 68) |
| PK626 | PB | Okara | 190 (139 – 250) | 104% (79 – 143) |
| PK627 | PB | Pakpattan | 222 (162 – 292) | 98% (75 – 135) |
| PK628 | PB | Rahim Yar Khan | 300 (218 – 394) | 55% (42 – 76) |
| PK629 | PB | Rajanpur | 284 (207 – 373) | 49% (37 – 68) |
| PK630 | PB | Rawalpindi | 184 (134 – 242) | 76% (58 – 104) |
| PK631 | PB | Sahiwal | 216 (157 – 284) | 79% (60 – 109) |
| PK632 | PB | Sargodha | 248 (181 – 327) | 107% (82 –147) |
| PK633 | PB | Sheikhupura | 257 (187 – 338) | 55% (42 – 76) |
| PK634 | PB | Sialkot | 211 (153 – 277) | 90% (69 – 124) |
| PK635 | PB | Toba Tek Singh | 251 (183 – 330) | 71% (54 – 97) |
| PK636 | PB | Vehari | 244 (178 – 321) | 78% (59 – 107) |
| PK701 | SD | Badin | 436 (318 – 573) | 38% (29 – 52) |
| PK702 | SD | Central Karachi | 121 (88 – 160) | 92% (70 – 127) |
| PK703 | SD | Dadu | 371 (270 – 487) | 32% (25 – 44) |
| PK704 | SD | East Karachi | 124 (91 – 164) | 56% (42 – 76) |
| PK705 | SD | Ghotki | 346 (252 – 455) | 38% (29 – 52) |
| PK706 | SD | Hyderabad | 200 (146 – 263) | 91% (69 – 125) |
| PK707 | SD | Jacobabad | 388 (283 – 510) | 36% (28 – 50) |
| PK708 | SD | Jamshoro | 407 (297 – 535) | 67% (51 – 92) |
| PK709 | SD | Kambar Shahadad Kot | 271 (197 – 356) | 35% (27 – 49) |
| PK710 | SD | Kashmore | 425 (310 – 559) | 24% (18 – 33) |
| PK711 | SD | Khairpur | 462 (337 – 607) | 28% (21 – 38) |
| PK712 | SD | Korangi Karachi | 121 (88 – 159) | 82% (63 – 113) |
| PK713 | SD | Larkana | 236 (172 – 310) | 72% (55 – 99) |
| PK714 | SD | Malir Karachi | 202 (147 – 265) | 45% (34 – 62) |
| PK715 | SD | Matiari | 387 (282 – 509) | 39% (30 –54) |
| PK716 | SD | Mirpur Khas | 324 (236 – 426) | 61% (46 – 84) |
| PK717 | SD | Naushahro Feroze | 350 (255 – 461) | 43% (33 – 59) |
| PK718 | SD | Sanghar | 419 (305 – 551) | 39% (30 – 54) |
| PK719 | SD | Shaheed Benazirabad | 436 (317 – 572) | 54% (41 – 74) |
| PK720 | SD | Shikarpur | 407 (297 – 535) | 30% (23 – 42) |
| PK721 | SD | South Karachi | 125 (91 – 164) | 134% (102 – 184) |
| PK722 | SD | Sujawal | 428 (312 – 562) | 26% (20 – 36) |
| PK723 | SD | Sukkur | 281 (205 – 370) | 65% (50 – 90) |
| PK724 | SD | Tando Allahyar | 418 (305 – 550) | 22% (17 – 30) |
| PK725 | SD | Tando Muhammad Khan | 439 (320 – 577) | 41% (32 – 57) |
| PK726 | SD | Tharparkar | 408 (297 – 536) | 25% (19 – 34) |
| PK727 | SD | Thatta | 419 (305 – 550) | 20% (15 – 28) |
| PK728 | SD | Umer Kot | 398 (290 – 522) | 71% (54 – 97) |
| PK729 | SD | West Karachi | 145 (106 – 190) | 43% (32 – 59) |

AJK: Azad Jammu & Kashmir; BA: Balochistan; GB: Gilgit-Baltistan; ICT: Islamabad Capital Territory; KP: Khyber Pakhtunkhwa; PB: Punjab; SD: Sindh.
