# Supplementary material for: Subnational tuberculosis burden estimation for Pakistan
Source: PLOS Glob Public Health. 2024 Sep 23;4(9):e0003653. doi: 10.1371/journal.pgph.0003653 (PMC11419375; doi:10.1371/journal.pgph.0003653)
Supplement: Attachment — Submitted filename: Response to Reviewers.docx [file pgph.0003653.s004.docx]

**Response to reviewer comments**

We thank the journal and the reviewer for their constructive review of our work. We have revised the manuscript accordingly. Please find the responses below:

**Reviewer 1:**

1. **Line 181 – The authors suggestion that the CDR over 100% suggests patients crossing district lines to access care seems more appropriate for the discussion section than the methods.**

Thank you for the suggestion. We agree that this is an interpretation of the results and thus we have moved it to the discussion.

2. **Line 225 – How do the authors distinguish between under-performance and cross-district health utilization when it comes to districts with low CDRs? Low CDRs may represent lower than expected notifications (due to undiagnosed TB or cross-district care seeking) but could also reflect overestimates of the true prevalence. Given the limitations of the data used in the model (especially using subnational prevalence data that is over a decade old – as this data may not reflect the current situation), I think the authors should discuss this possibility.**

Thanks for bringing this to our attention. We thoroughly agree that districts with low CDR represent a mismatch between model estimates and empirical notification data from the NTP. From this perspective, we made explicit statements of this: “*Foremost, this could reflect a mismatch between empirical notification data and model estimates that emerge based on methods and assumptions made*” (Line 252-254). Away from model performance and placing the results into context, we can interpret low CDRs as either under-performance of the district to detect all individuals with TB (as noted in lines 254-257) or, when placed next to a district with over-reporting of the CDR, as cross-district health utilisation (as noted in lines 249-250).

3. **The authors do not discuss the relative importance of the variable selection process. What are the implications, and how might the results vary with different variables or different RRs? A sensitivity analysis may be warranted here.**

Thanks for raising this. The variable selection process is important as district distribution is performed based on weighted scores generated from the variables. The principle of SUBsET is to maximise differentiation between the geographical areas, and therefore includes many variables to for which the required data is available and has endorsement of the NTP. The challenge is usually too few variables, rather than having to choose. We have performed a sensitivity analysis where we included one of the variables only, highlighting which variable contributed the strongest (indicated in line 156-159 of the Methods section). This is in line with the sensitivity analysis performed by Parwati et al. for the iteration of the SUBsET model in Indonesia. The results from the sensitivity analysis and a figure showing a comparison of TB incidence estimates per district under each model have now been added to the Results section (Line 209-220 and Figure 5).

4. **The authors clearly describe the use of the SUBsET tool and the resulting output from the model. The accessibility and ease of use of an Excel-based tool is convincing. However, the authors do not appear to discuss what this implementation might look like; given the strong collaboration with the NTP, it would be nice to see more specific ideas for how this tool might be used, beyond simply running the model and targeting resources to high-burden or underreporting areas, which can be done with any model.**

We thank the reviewer for their comments. We have opted to expand a section to describe how implementation of the model might look like: “*Despite its limitations, the SUBsET tool generates subnational estimates of TB burden through a transparent and accessible approach. Developed in collaboration with the NTP at each stage, the tool is well-informed by both data and policymakers, as with previous iterations. The NTP provides most of the input data and advises on which variables should be included for burden distribution. Additionally, the tool’s Excel-based platform makes it accessible to non-modellers by clearly presenting its functionality. The final version of the tool is designed to be dynamic, allowing for updates and improvements as new data become available*” (Line 295-303). Here, we argue that SUBsET is unlike other models as it allows for future iterations at the will of the NTP. As a note, via personal communication with Prof Rein Houben who was involved in the previous application of SUBsET in Indonesia, this model is still being used by the NTP as part of their communication and discussions with subnational partners.

5. **Data availability is a key limitation, and this is well-noted by the authors. However, it is difficult the see the utility of this, or any model, without reliable and timely data. Can the authors please clarify how they envision the use of this model in a programmatic setting, given that relying on old prevalence surveys may be providing out-of-date information?**

This is good point raised by the reviewer. Data availability is a key limitation. We are using a dated TB prevalence survey (2010-11) as a first step to distribute TB burden by region. We argue that while an update survey would be expected to reflect a lower TB prevalence, “*regional distribution is unlikely to change significantly due to generational stability of sociodemographic determinants within regions*” (Line 269-270). Furthermore, we assert that the model’s design allows for a simple updating of the model inputs: “*in contrast to other models, the simplicity of the model allows for future adaptation, making estimates readily available after new surveys are conducted*” (Line 271-272). This design allows for the inclusion of additional variables if data become available.

6. **I think it would be helpful to have more discussion comparing the findings of other subnational estimates – where are the discrepancies noted? Are there certain regions or contexts where estimates seem the most reliable (or unreliable)? Do different models produce consistently higher estimates than other? What other differences between model outputs are notable? While I understand the challenges in formal validation of these model outputs, it would be helpful to have a more specific understanding of how they compare in order for readers to determine whether this model is more or less appropriate than others.**

Thanks for this suggestion. We have carefully considered it during both the initial manuscript writing and after receiving reviewer comments. However, we believe that the proposed additions do not significantly enhance the study. Firstly, there are major differences in the estimation method, with some of the model incorporating TB notifications into their calculations. SUBsET does not do this, as explained in the manuscript, but rather uses them as comparison. Secondly, formal comparison of the models from Alba et al. is beyond the scope of this paper. We have added a line in the paper to reflect this: “*While a formal comparison with the model outputs described in Alba et al. is beyond the scope of this paper, we subscribe to the main conclusion that substantial differences are likely to exist*” (Line 241-243).

Additionally, the authors would like to express their commitment to making all data available and remind the reviewers that a GitHub link is provided where the data and updated analysis code can be found.

Please let us know if we can provide any additional information. We look forward to hearing from you at your earliest convenience.

Warm wishes,

Alvaro Schwalb, for the authors
